# Review of the literature on combined oral contraceptives and cancer

**DOI:** 10.3332/ecancer.2022.1416

**Published:** 2022-06-23

**Authors:** Mustafa Kamani, Utku Akgor, Murat Gültekin

**Affiliations:** Department of Obstetrics and Gynaecology, Hacettepe University Faculty of Medicine, Ankara 06230, Turkey

**Keywords:** combined contraceptives, hormonal contraception, breast cancer, ovarian cancer, cervical cancer, liver cancer

## Abstract

Millions of women have given preference to the use of combined oral contraceptives (COCs) since its introduction in the 1960s. Both oestrogens and progestogens can regulate proliferation and it is plausible these effects may contribute to carcinogenesis. We aimed to review the accumulated knowledge to date to appreciate the modifying effects combined oral contraceptives may have on carcinogenesis. Our methodology involved a review of the current published literature, paying attention to studies published in the last 20 years. It has been noted that the overall cancer odds do not change with the use of COCs. Increased risk for breast cancer with COC use is not consistently backed in the literature; the results range from no increase in risk to a 20%–30% elevation in risk, and the risk seems to be temporary, limited to recent or current regular COC use. Also, diagnosed breast cancer cases seem to be clinically advanced in ever-users compared to never-users. Data show that the ongoing and prolonged use of COCs may provide diminished risk for endometrial, colorectal and ovarian cancers. Although studies do not clearly support increased risk with COC use in high-risk groups, such as women with family history of cancer or BRCA carriers, local and international guidelines are available for clinical decision-making. For cervical cancer, COCs seem to enhance the risk with more than 5 years of use, and in many studies, this enhanced risk diminishes after discontinuation and restores to those of never-users within 10 years. The relationship between COC use and liver malignancy risk assessments has provided conflicting findings. Some studies have suggested that hormonal contraceptives may increase the risk of not only hepatocellular carcinoma but also intrahepatic cholangiocarcinoma. Combined oral contraceptives are safe and effective and the effects are reversible. Patients who pursue family planning should be warned of possible carcinogenic outcomes, but it should also be explained that—in addition to sexual health advantages—preferring COCs may also decrease the risks of endometrial, colorectal and ovarian cancers.

## Introduction

Combined oral contraceptives are classified by the World Health Organisation among Group 1 carcinogens for their possible relationship to liver cancer, *in situ* and invasive cancer of the uterine cervix and breast cancer [1]. From the time of its addition to the contraceptive arsenal, millions of women have used COCs, oftentimes for lengthy durations and at a period of well-being. Since the development and approval of the first COC pill, formulations have evolved from comprising high-dose oestrogen (150 mcg) to very low doses (10 and 20 mcg). Although there is a wide array to choose from, and there are many doses accessible, all consist of a progestogen and an oestrogen. Pregnancy is averted fundamentally via blocked ovulation. In addition, alterations to the endometrium and cervical mucus are also important factors preventing pregnancy [2]. The users were especially concerned about cancer, given the widespread usage and frequent media scares. Both oestrogens and progestogens can regulate cell growth and reproduction, and it is plausible that this proliferative influence may contribute to carcinogenesis in susceptible tissues. The aim of this review was to analyse the available up-to-date literature regarding malignancy risks linked to the use of COCs. The search was performed in the PubMed database for oral contraceptive/combined oral contraceptives/contraceptives/contraception in title or abstract and cancer in title. A total of 2,483 papers were returned. Titles of the last 20 years were the primary focus and studies published in high-impact journals, which have large cohort sizes and high citation counts, were selected for more in-depth narrative reviews.

## Overall cancer risk

Up to now, there have not been any studies showing that overall cancer risk or mortality related to cancer in general has increased among COC users. Cohort studies are notably advantageous when it comes to analysing the general proportion of risks and advantages related to a COC exposure. Absolute risk of mortality from cancer was assessed in COC users in three large cohort studies, which are the Royal College of General Practitioners (RCGP) study, the Nurses’ Health Study and the Oxford Family Planning Association research; between ever-users and never-users, none of these studies found significant discrepancy [3–5]. Overall, cancer possibility does not increase with COC use. The evidence proposes that ongoing users of COCs may have elevated odds of malignancy of the liver, cervix and breast in comparison with non-users. The majority of studies propose that the elevated risks of mentioned malignancies deteriorate subsequently after cessation of COCs, restoring within about 10 years to that of non-users. On the other hand, ongoing and prolonged use of COCs provide diminished risks for ovarian, endometrial and probably colorectal cancers. This protection appears to remain for years after cessation of COCs, as it may exceed 30 years. Long-standing cancer benefits might neutralise the interim adverse effects on the assumption that they persevere until later in life because the majority of malignancies become frequent after 50 years of age.

Long-term malignancy risks and benefits were analysed in the RCGP oral hormonal pregnancy prevention study, which covered a cohort of more than 46,000 women who were followed up as far as 44 years [6]. In this study, it is reported that patients who had used COCs are not in danger of new malignancy risks later in life. Regular COC use may have a protective effect from ovarian, colorectal and endometrial malignancies, which are likely to continue many years after cessation, possibly more than 35 years in the cases of ovarian and colorectal cancer. Enhanced risks for cancer of the breast and cancer of the uterine cervix that are observed in recent and ongoing users are stated to be temporary, diminishing after 5 years of cessation to those of never-users.

Long-term cancer risks were evaluated in a cohort of 267,400 female textile workers in China; no increased risk of cancers combined or of thyroid, liver, breast, lung, ovarian, colon, pancreatic, gall bladder, rectal, cervical or stomach cancers were detected. Only cancers of the uterine corpus were found to be reduced with COC use [7].

## Breast cancer

**Figure 1.**
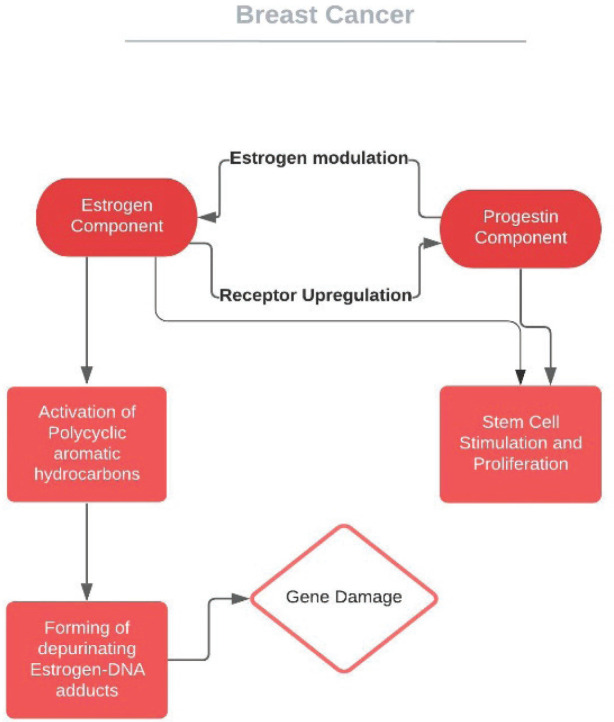
Pathways correlating the effect of COCs on breast cancer.

Oestrogens and progestogens are among the modifiable factors that may increase the risk of breast cancer (BC) and this has been confirmed, for instance, in hormone replacement treatment (HRT) in the post-menopausal population. From the time of its introduction, a similar proposal has been suggested for COCs. In the breast, both oestrogen and progesterone have a proliferating effect, probably due to stem cell stimulation [8]. Inherent oestrogens (oestradiol and oestrone) are suggested mutagens over carcinogens by way of a gene damaging mechanism [9], whereas the mediating route of progestin is more complicated [10, 11] (Figure 1b). Scepticism remains regarding the association between the consumption of COCs and the risk of BC. Researches of BC risk among women who use COCs show conflicting results: from no increase in risk to a 20%–30% elevation in risk. The majority of articles have categorised participants in accordance to whether they were ongoing, recent or previous users of COCs or if they had ever used COCs. The findings of many epidemiologic studies state no association. Any effect seems to be temporary or limited to recent or ongoing COC usage (Table 1).

In the Nurses’ Health research, the RCGP research and the Oxford Family Planning Association research, which are three large prospective cohort studies, neither long-lasting past COC use nor ongoing use was linked to an elevated risk of BC [5, 14, 17].

In a population-based, case–control study, which consisted of more than 4500 women with breast cancer, BC risk did not vary significantly between ongoing (relative risk (RR) 1.0, 95% CI: 0.8–1.3) or previous COC users (RR 0.9, 95% CI: 0.8–1.0) [15]. The RR did not arise persistently with long time use or with larger doses of oestrogen. For women with a family history of BC, the use of oral contraceptives was not associated with an elevated risk of breast cancer nor was the initiation of COC use earlier in life. Risk does not seem to be varied with different COC formulations [18].

On the contrary, some articles have proposed a relationship between COC use and BC [6, 19–21] (Table 2). The results are diverse regarding the aforementioned categories of use. It is not known if this relationship is a biological effect or an outcome of increased diagnosis, whether different COC doses might have varying effects or a combination of reasons. However, the International Agency for Research on Cancer (IARC) Working Group on the Evaluation of Carcinogenic Risks to Humans released a monograph in 2007 and judged that there was adequate documentation to demonstrate the carcinogenicity of COCs in humans, with an elevated risk of BC in patients who were using them or had used them recently. Even so, if we inspect these articles that associate COC use with BC, the absolute risk is very low.

In a meta-analysis by the Collaborative Group on Hormonal Factors in Breast Cancer consisting of 54 studies in 25 countries and including over 53,000 women, COC use was related to an elevated risk of BC (RR 1.24, 95% CI: 1.15–1.33), which faded after cessation (RR 1.16 after 1–4 years, RR 1.07 after 5–9 years) and ceased after 10 or more years [19]. The length of COC use and the variety of COCs had no consequence on BC risk after adjusting for time of use and the malignancies detected then are less advanced clinically than the malignancies detected in never-users.

1.8 million women from a Danish registry were followed up for an average of 11 years in a prospective cohort study. In this research, ongoing or late COC users displayed an elevated risk of BC in contrast to counterparts who never gave preference to COCs (RR 1.19, 95% CI: 1.13–1.26) [21]. Derived risk for any hormonal contraception in comparison with non-users (RR 1.20, 95% CI: 1.14–1.26) was akin to this risk and rose with a longer period of usage. The overall rise in detected BC cases in the COC user group was small: about 1 added diagnose per 7,690 women annually. For women most likely to use COCs, who are below the age of 35 years, the overall enhanced risk was 1 added diagnosis per 50,000 women annually.

Findings from the Nurses’ Health Study, which is the largest cohort to date [22], including 121,577 women, reported that oral hormonal contraception usage is connected to elevated mortality due to BC for participants who have used OCs for 5 years or more in contrast with participants who never used COCs (RR, 95% CI: 1.26 (1.09–1.46)). In 2021, the Nurses’ Health Study II prospective cohort reported a higher risk for current users (HR 1.31; 95% CI: 1.09–1.58) compared to never-users. For former users, after 5 years of cessation, the risks were similar to those of never-users (23). However, the majority of studies do not show any association [17, 24–28].

In a recent meta-analysis, COC use and BC risk showed a significant linear dose–response relationship; 0.7% elevation in risk was reported with every 1 year increase of age [29].

Since there is no consensus in the literature on whether breast cancer is associated with COC use, it is wise to consider the details if the latter is true. Diagnosed breast cancer cases were less clinically advanced in ever-users compared to never-users [19]. Additionally, varying COC formulations may comprise varying risks of malignancy; continuing observations of these relationships may produce new information as COC formulations evolve. The RR of 1.2 (20% increase in risk) reported by Mørch [21] similar to that found by the Collaborative Group on Hormonal Factors in Breast Cancer [19] where the risk of breast cancer was 1.24 and comparable to preceding prospective studies [20, 30]. From this, it can be deduced that the malignancy risk for breast cancer with contemporary formulations is akin to the risk of older ones.

Combined oral contraceptives are not recommended for women with a personal history of BC (United States Medical Eligibility Criteria (MEC) category 4 (unacceptable risk) for current breast cancer, category 3 (risks outweigh the benefits) for past and no evidence of disease for 5 years) [31]. However, evidence [15, 19, 32–49] does not show an elevated risk for BC among women with either a family history of BC or BC susceptibility genes; therefore, women with breast cancer susceptibility genes (such as *BRCA*) or a family history of BC may use COCs at least for short term durations. Beside the point in a meta-analysis by Moorman *et al* [50], studying COC use among *BRCA1* and* BRCA2* mutation carriers, COC use had an elevated but non-statistically significant relationship with BC (odds ratio 1.21, 95% CI: 0.93–1.58).

## Ovarian cancer

Ovarian cancer is the fifth leading cause of cancer death and the eight most common cancer in women [51]. Despite the progress in initial management, the rate of death for ovarian cancer continues to be uppermost among gynaecological cancers. Since ovarian malignancies characteristically appear at a more advanced stage clinically (with a parallel greater rate of death) than other frequent malignancies [51], there has been passionate concern in developing successful screening strategies. Unlike breast cancer, screening studies for ovarian cancer so far have not exhibited decreases in the rate of death and false-positive rates have been excessive [52–58].

COCs show a likely promising initial preventive measure for ovarian cancer. Researches have consistently demonstrated that continued use of COCs lowers the risk of ovarian cancer. Various broad pooled studies advocate that COCs provide a protective effect on ovarian malignancy risk, with a risk decrease of up to 50% with longer periods of COC use [59–62]. The largest pooled analysis today predicts that oral hormonal contraceptive pill use already has prevented 200,000 incidents of ovarian malignancy and 100,000 deaths from this cancer worldwide [60].

In a meta-analysis, which pooled 24 studies of oral hormonal contraception as primary protection for ovarian malignancy, ever-use was correlated with a decrease in ovarian malignancy in comparison with never-use (OR = 0.73, 95% CI: 0.66–0.81), with more than 50% decrease among participants with ≥10 years of usage [14]. Relationship between the degree of the protective effect and duration of COC use is correlated. The registry of this research consisted of both women who desired contraception and women who were pursuing a decrease in their possibility for ovarian malignancy. Separate meta-analyses have found comparable advantages in women at high risk for ovarian malignancy [15, 16].

In the aforementioned reanalysis by a collaborative group, risk reductions per 5 years of COC use were different for different histological subtypes; for serous, 22.1%; for endometrioid, 27.1%; for clear cell carcinomas, 21.3%; and lastly, 6.7% for mucinous tumours, which was non-significant [60]. Similar preventive ratios are obtained with tubal ligation; most reduction in endometrioid subtypes, less marked for serous neoplasms and for mucinous tumours, nil [63]. This is in concordance with hypothesises that suggest primary ovarian mucinous tumours are originated from germ cells [64] or Walthard cell nest [65], accordingly being less vulnerable to contraceptive or reproductive effects.

To understand the underlying mechanisms for how COCs prevent ovarian cancer, the process of ovarian cancer itself should be evaluated foremost. However, the aetiology of epithelial ovarian cancer has been debated for over more than half a century. It is known that ovarian cancer may develop through mutations with step-wise fashion (type 1, low-grade pathway) or much more aggressive mutations without precursor lesions causing widespread metastasis (type 2, high-grade pathway) [66]. Carcinogenesis theories assumed the coelomic epithelium of the ovary was the primary source for cells undergoing malignant degeneration [67–70]. It has been suggested that following every ovulation,

damage to the flat mesothelium was ending with entrapment of its cells during the healing process. Undergoing Müllerian metaplasia, they gave rise to mucinous, serous and endometrioid cystadenomas, which are cancer precursor lesions. But perplexities are born on the coelomic ancestry; most encountered mucinous, serous and endometrioid types are identical to intestinal/endocervical, fallopian tube and endometrium carcinomas, morphologically. Moreover, Müllerian cells are not present in ovaries. In addition to this, studies have failed to show a coelomic precursor lesion for these cancers, and therefore epithelial ovarian cancers are accepted as *de novo* [71].

**Figure 2.**
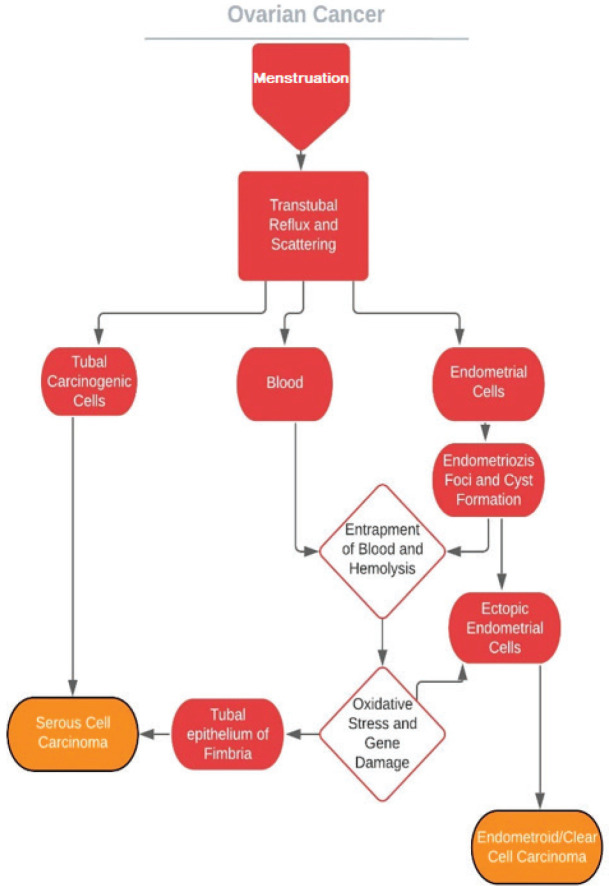
Probable mechanisms of ovarian cancers of Mullerian orgin.

Novel theories propose that the plantation of tubal and endometrial cells on ovary epithelium is the initial step for the process for these cancers of Müllerian origin [65, 72, 73] and retrograde menstruation provides the carcinogenic iron [74]. These theories are in concordance with the high prevalence of coexisting serous tubal intraepithelial carcinomas (STIC) in both BRCA1 and BRCA2 carriers undergoing risk-reducing salpingo-oophorectomy [75–80] and in women with non-hereditary high-grade serous ovarian cancers [81–83]. In addition to this, the relationship between clear cell/endometrioid cancers and endometriosis has been demonstrated [84–90]. By way of reduced tubal secretion and motility, ovulation inhibition, menstrual flow decreases and endometrial gland atrophy COCs may hamper the aforementioned pathways (Figure 2).

## BRCA1/2 carriers

There has been uneasiness that oral hormonal pregnancy prevention might elevate the risk of BC in susceptible populations like mutation carriers. Today, all women who are susceptible to gene mutation are proposed risk-reducing salpingo-oophorectomy (RR-BSO) at an age before which they are at the highest risk for ovarian malignancy as a routine practice; thus, COCs as protective measures are not required. Prophylactic surgeries are the standard of preventive care for these individuals. However, for women who must use COCs for their hormonal diseases or who have decided against RR-BSO, data advocate a decreased risk of ovarian malignancy in women with mutated susceptibility genes who take COC pills, although the hypothetical risk of elevated BC continues to remain. As discussed earlier, this risk was suggested by many studies but has not been consistently backed in the literature.

Women who possess the BRCA1 and BRCA2 genes compose a high-risk population and correspond to 10–15% of all BC cases. In a case–control study which consisted of nearly 2500 matched pairs of women who carried BRCA1 gene, oral hormonal contraception usage was related with an elevated risk of early onset BC, granted that medication commenced under the age of 20 (OR = 1.45, 95% CI: 1.20, 1.75) [91]; and the risk built up by 11% for each added year of use.

18 comparative, retrospective studies of oral hormonal contraceptive use in BRCA1/2 mutation carriers were analysed. This meta-analysis included 1503 cases of ovarian malignancy and 2855 cases of BC [92]. The results demonstrated that ever-use of oral contraceptives was related to a decreased risk of ovarian malignancy (RR 0.50, 95% CI: 0.33–0.75); this finding did not differ between mutation groups. A longer period of use raised preventive power. In 2021, this effect was also backed in a cohort [93]. The largest data in the analysis, a case–control study that included 798 women with ovarian malignancy, showed a 5% reduction in the risk of ovarian malignancy per year of COC use [94]. Moreover, COC use seems to be correlated with a reduced risk of fallopian tube malignancy in the common population [95].

In the study outlined above, there was no indication of a significantly elevated BC risk in COC users in general, for ongoing users or in the first decade after discontinuance of use [92].

In a meta-analysis deliberated beforehand, which studied COC use among BRCA1/2 mutation carriers, relationships between ever-use of oral contraceptives and ovarian and breast cancer among women who are carriers are akin to those shown for the common populace [50].

In a recent study, a prospective cohort study which comprised 6030 BRCA1 and 3809 BRCA2 carriers, retrospective and prospective analyses were inconsistent. In the prospective arm of BRCA1 carriers, there was no elevation in the risk of BC, but retrospective results were consistent between full-cohort (HR: 1.39) and left-truncated (HR: 1.26) analyses. The BRCA2 carriers’ prospective arm and full-cohort showed elevations in the risk of BC (HR: 1.75 and HR: 1.52, respectively) but retrospective results were inconsistent between full-cohort and left-truncated analyses (HR: 1.06) [96].

In a recent systematic review, overall data on BRCA mutation carriers were found to be very limited to decide the use of COCs in this population. BRCA1/2 mutation carriers also benefitted from the protective effects of COC use, which decreases the risk of ovarian cancer [97]. On the contrary, a new meta-analysis reported the elevated risk for breast cancer among long-term users (>5 years), while a protective effect for ovarian cancer was observed regardless of COC use [98]. Therefore, an elevation in BC risk cannot be ruled out. At any rate, cancer risk for ovaries is still raised up under COC use in this population and RR-BSO is favoured at a reasonable age [97].

## Cervical cancer

GLOBOCAN predicted 570,000 cases and 311,000 deaths for cervical malignancy in 2018 and these numbers rank it to be the fourth most frequently diagnosed cancer and the fourth leading cause of cancer death in women [99].

Human papillomavirus (HPV) is the essentially fundamental (but not enough) factor of cervical malignancy [100], which is classified as a group 1 carcinogen by the IARC working group with its 12 subtypes [101]. Immunosuppression (particularly HIV), tobacco use, parity and oral hormonal contraception use are other significant factors [102]. A far-reaching review overseen by IARC, as mentioned earlier, classified the use of COCs as carcinogenic to humans, and this was established on the stated relationships with cervical malignancy in part [103]. There is concern that HPV carriers may be at a notable risk for cervical malignancy [104]. Notwithstanding, screening for cervical malignancy should not be a precondition for the planning of pregnancy prevention strategies [105].

The age-specific incidence rate of cervical cancer increases after 25 years of age and peaks around 40 years in high-resource countries, although it continues to rise until 55–69 years of age in low-resource countries and so the contribution of COCs to the life-long incidence of cervical malignancy will revolve around on mostly the consequences later in life, when majority are former users. Long-term cervical malignancy risk does not seem to scale up among ever-users in comparison to never-users (incidence rate ratio 1.31, 99% CI 0.84–2.04) [6]. Yet, lamination by recency of COC use shows that ongoing or recent use (<5 years) is accompanied by elevated risk of cervical malignancy (incidence rate ratio 2.32, 99% CI 1.24–4.34) [6]. The reported risk seems to wane after relatively 5 years of ceasing COCs, with no indication of malignancy appearing at heightened risk in ever-users later in life.

In the EPIC study, which consisted of a cohort of over 300,000 participants who were followed up for 9 years, the relationship between hormonal cofactors and risk of acquiring cervical malignancy was assessed [106]. Similar to comparable studies, it has been found that cervical malignancy risk increased with continuation of use and waned after discontinuation, and the study cemented the acknowledged firm correlation between oral contraceptive use and cervical pre-cancer and cancer.

In a recent cohort study conducted in Denmark, nearly two million women of reproductive age were evaluated. Increased risk of cervical cancer was observed among recent and current users of COCs (RR 1.40 (95% CI: 1.28–1.53)). Risk analysis was similar for both adenocarcinoma and squamous cancer. This effect was observed more with the longer duration of use and declined after discontinuation. In this cohort, most women were unvaccinated against HPV; therefore, currently used COCs may have a similar risk profile compared to older formulations [107].

In a meta-analysis, which consisted of 24 studies comprising almost 17,000 patients with cervical malignancy and approximately 36,000 controls support this effect, the risk of cervical invasive malignancy was elevated among ongoing users of COCs with a prolonged period of use (RR ≥5 years usage versus never, 1.90 (95% CI: 1.69–2.13)). This enhanced risk diminished after discontinuation and was restored to those of never-users within 10 years. An identical model of risk was detected for both invasive and *in-situ* malignancy, and in high-risk HPV carriers [108].

**Figure 3.**
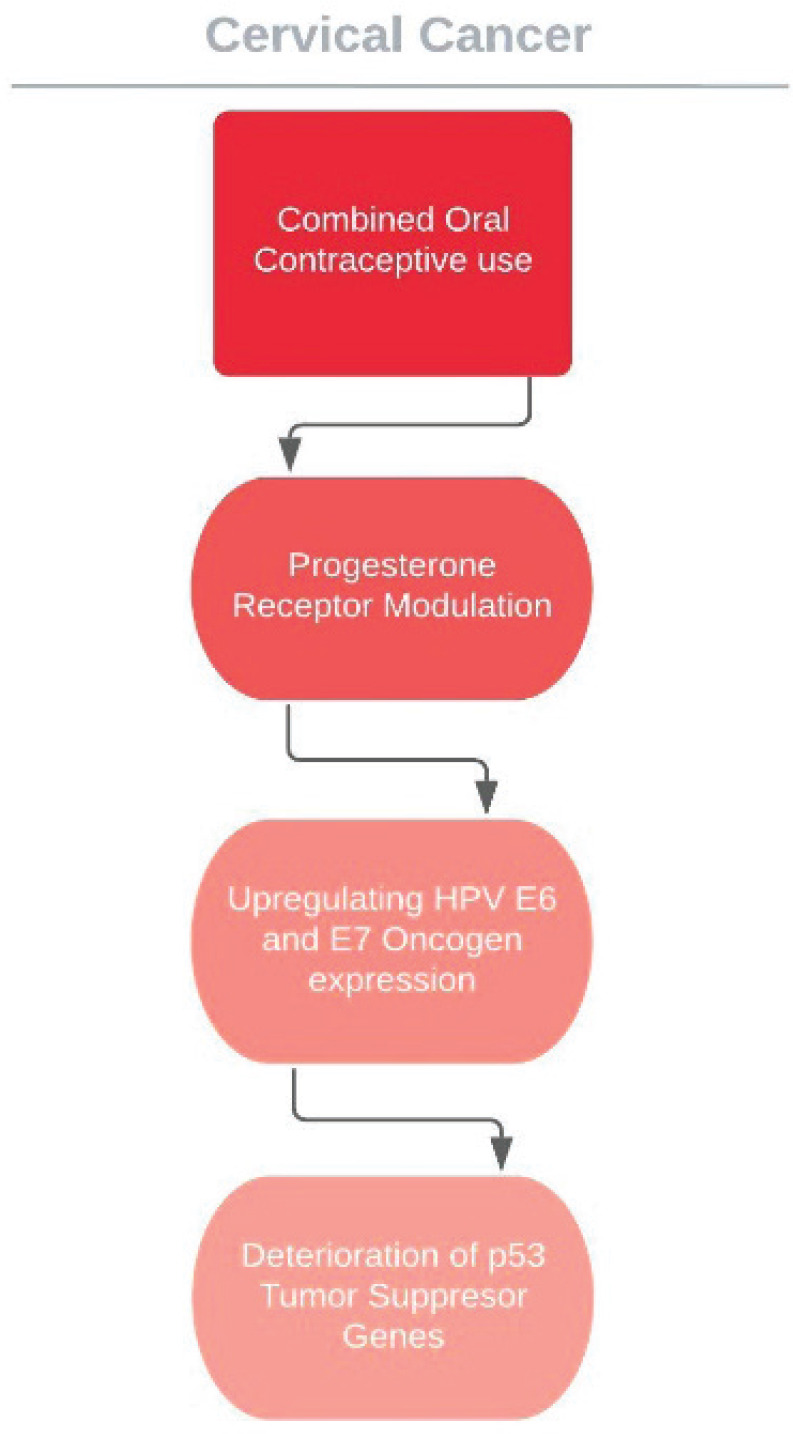
Suggested effect of COCs on HPV carcinogenesis.

The cervical cancer effects of COC usage is now known and elevated absolute risk in users is the valid measure of this consequence; however, it does not tell much about how exactly the HPV reliant carcinogenetic process is enhanced. COC use might, for instance, enhance the cervical vulnerability to HPV transmission and resultant infection or it might alter persistence or disposition of virus or the advancement or reversion of malign and pre-malign lesions. A plausible pathway to elucidate the relationships between oral contraceptive use and cervical cancer risk is that cervical tissue hormone receptors, especially progesterone, may be affected and the course of HPV may be altered. Particularly, hormones used in contraceptives are assumed to intensify the expression of HPV 16 E6 and E7 oncogenes, prompting the deterioration of p53 tumour suppressor genes and augmenting the capability of the viral DNA to mutate cells and promote neoplasticity [108–111] (Figure 3). Data from transgenic mouse models support the presumed pathway of oestrogen combined with HPV oncogenes inducing cervical carcinogenesis; however, these studies show that progesterone suppresses cervical carcinogenesis in mice [112, 113].

The latest 2015 WHO guidelines for MEC for contraceptive use categorises the recommendations into four groups. For HPV positivity, cervical intraepithelial neoplasies and cervical cancer awaiting treatment, the WHO recommendation group is 2; the advantages of usage outweigh the risks [114]. While using COCs among women with persistent human papillomavirus (HPV) infection and CINs, patients should be reminded that longer (>5 years) durations of use may increase the risk of carcinoma *in situ* and invasive carcinoma.

## Endometrial cancer

The use of COCs provides prevention against endometrial neoplasia. Consistently, studies have found that the longer the period of usage, the more significant is the decrease in the risk of endometrial neoplasia. On average, every 5 years of use was related to a RR of 0.76; consequently, after approximately 10–15 years, the risk lowers by 50%. After discontinuation, the preventive influence goes on beyond 30 years, and does not give the impression to rely much on the oestrogen dose in formulations or individual traits like parity, body mass index or menopausal condition [115]. Since the rate of new cases of endometrial malignancy increases strongly later in life, the communal consequences of this converse relation lean primarily on the degree to which the decreased probability of endometrial malignancy carries on after discontinuation.

The decrease in cancer rate related to COC use appears to vary by malignancy form, being more effective for carcinomas (RR 0.69, 95% CI: 0.66–0.71) than sarcomas (0.83, 0.67–1.04; case-case comparison: *p* = 0·02)[115].

Findings from recent (2021) The Nurses’ Health Cohort Study II, including 107,069 women, showed oral hormonal contraception usage is connected to lower endometrial cancer risk (ever use, HR 0.77 [95% CI 0.65-0.91]; >10 years of use, 0.43 [0.32-0.58] vs. never OC use) [116]. These findings are consistent with previous meta-analysis and cohorts involving women who may have been exposed to higher dose formulations.

**Figure 4.**
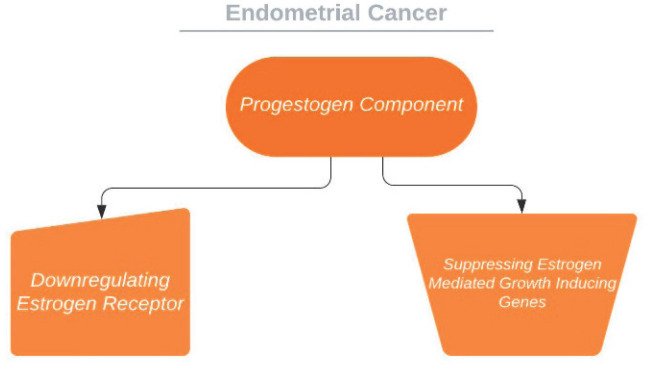
Suggested effect of progestogens on endometrium.

The use of COCs reduced the risk of endometrial cancer by 30%–40% in broad epidemiologic studies, and the risk decrease persevered for years after cessation [6, 115]. This advantage of hormonal contraception, which is assumably accurate for non-oral combined hormonal contraceptives as well, is thought to be mediated through progestogen ingredient which restrains endometrial proliferation and promotes differentiation. Progestin-only contraceptives seem to grant even stronger protection versus carcinogenesis of endometrium in epidemiologic studies [117–119]. On the other hand, in more former researches, COCs had larger doses compared to present-day formulations, which are progestogen prominent and hence may have preventive influences proportionate to progestogen-only preparations. This effect may be mediated through diminished exposure to unopposed oestrogen in the follicular phase, down regulation of oestrogen receptors and suppression of oestrogen mediated proliferation genes [120–122] (Figure 4). Moreover, it has also been shown that in women receiving perimenopausal HRT, the addition of progestins to the regime decreases the oestrogenic side effect on endometrial carcinogenesis [60, 123–125].

## Colorectal cancer

Colorectal cancer (CRC) is another common cancer in women, with relatively good survival rates that are at around 65%. The number of studies reporting on the relationship between COCs and CRC risk are voluminous and IARC has noted that the use of COCs may decrease the risk of colorectal cancer. For the past 20 years, still, there is no consensus between the results of epidemiological studies.

In a meta-analysis, which consisted of 23 studies comprising 14 case–control and 9 cohort studies, relative CRC risk for ever-use versus never-use was 0.8 [126]. In this study, the relationship between duration and risk was not evaluated but recent use was found to be more protective (OR = 0.7).

In another meta-analysis, which consisted of 29 studies comprising nearly 16,000 colorectal cancer cases, relative CRC risk for ever-use versus never-use was 0.8 [127]. In this study, the duration of COC use was inversely associated reduction of risk and effect was not dose-related. In the Royal College of General Practitioners’ Oral Contraception study, protection from CRC was presumed to be longer than 35 years [6].

In a recent population-based case–control study conducted in Northern Israel, nearly 3,000 CRC cases were evaluated. It was found that COC use was very inversely associated with CRC risk among people with Jewish and Arabic origin (ever-use versus never-use; odds ratio: 0.49 (0.39–0.62) and 0.14 (0.04–0.47), respectively) [128].

However, in some recent high-quality cohort studies, including the Nurses’ Health Study, neither long-lasting past COC use nor ongoing use was linked to an elevated risk of CRC [6, 104, 129–134] (Table 3). However, the studies were not consistent in their findings with risk reduction with prolonged use in comparison to the findings on ovarian and endometrial cancers [104, 132, 133].

In a cohort of 1.3 million women, which was followed up for 13 years, ever-use of COCs was found to be associated with an elevation in risk of anal cancer (OR = 1.51, 95% CI: 1.24–1.83) [135]. This may be associated with HPV-related pathways as in cervical cancer.

**Figure 5.**
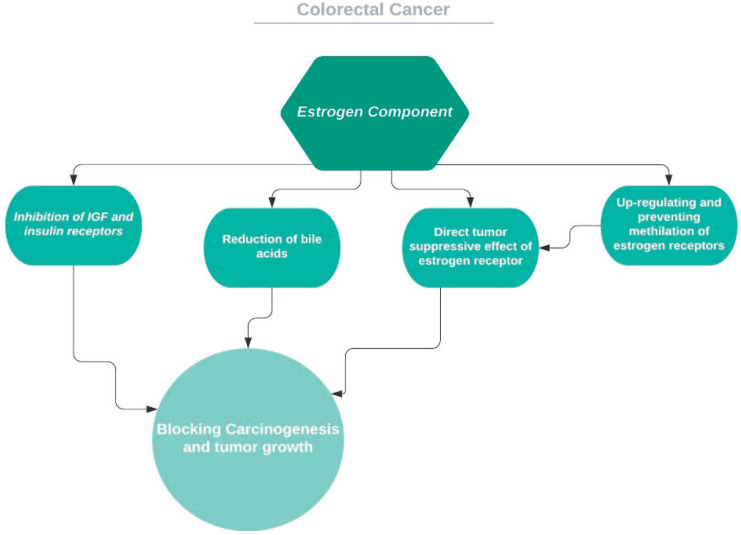
Proposed mechanisms for the effect of estrogen on colorectal cancer.

Several mechanisms, direct and indirect, were suggested for how COCs modify the risk for CRC [136] (Figure 5). Insulin-like growth factor regulation via oestrogen and body mass index is one of these suggestions [137]. Another would be reduction of secondary bile acids [138]: bile acids proliferative and carcinogenetic effects on colon cells were demonstrated in both rats and humans [139, 140]. Additionally, oestrogen has a direct growth-inhibiting effect on human colon cancer cell lines by way of its own receptor [141]. Oestrogen receptors are known to interact with different pathways detected in colon tumourigenesis [142, 143]. These tumour suppressive receptors are demonstrated to be diminished with age in colon tissue through methylation of the receptor gene and this gene may be upregulated by circulating oestrogen [144]. Even though more and more studies are being conducted on the mechanisms of cancer, such mechanisms are still waiting to be clarified.

COC use may decrease the CRC risk by at least 15% and this effect seems to not be dose-related, lasting for more than 30 years and not changing with duration of use.

## Liver cancer

Primary liver malignancy is the sixth most common diagnosed cancer and the ninth most common cancer in women, and it is mostly an issue in less advanced countries [99]. Hepatocellular carcinoma (HCC) is responsible for 70%–85% of primary live malignancy in majority of the regions [145].

The relationship between COC use and liver malignancy risk assessments has provided conflicting findings. In a meta-analysis of 12 case–control studies, which included over 700 women, the results relating to cancers, like HCC, are varied [146]. In one review, 6 of the studies showed between 2 and 20-fold increase in risk [146]; at the same time, a larger study revealed that COC use was not related to an increase in risk related to hepatic neoplasms [147].

Regarding the incidence of HCC, the effect of sex has been well demonstrated. Men are developing cancer more often than women, and this has brought about the hypothesis that female sex hormones may prevent hepatocellular carcinoma. Backing this, a case–control study, which consisted of 234 patients with treated HCC and 282 controls, demonstrated that post-menopausal HRT was decreasing the incidence of HCC (148). In a meta-analysis, it has been shown that people with differences in oestrogen receptor 1 gene had different odds of developing HCC (149). Furthermore, in a study which pooled nearly 800.000 women from 11 cohorts, surgical excision of both ovaries was significantly related to elevated odds of developing HCC (HR = 2.67; 95% CI: 1.22–5.85), after calculating for other individual and clinical cofactors and the length of HRT [150]. In the stated research, COC use was not related to an increase.

In a meta-analysis comprising 14 case–control studies and 3 cohort studies, a significant difference could not be detected; however, a detached investigation of these 14 case–control studies, contrary to the cohorts of the same meta-analysis, revealed a significant elevation in odds ratio (1.55) [147]. In a case-cohort investigation, which consisted of 267.400 female workers, 420 liver cancer cases were analysed with appropriate control group and odds ratio for COC use was not significant (95% CI: 0.82, 0.60–1.13) [7].

There is some evidence regarding the probable biochemical pathways through which hormones may promote carcinogenesis in liver. Hepatocytes carry oestrogen receptors, which are found to be upregulated in HCC, probably because of their proliferative and mutagenic effects [151, 152]. The increase in liver cancer remarked by IARC is not confirmed by the recent studies mentioned. Since IARC noted that the elevation of risk related to liver malignancy observed in regions is poor in cases of chronic liver disease and hepatitis B alone, it should be emphasised that these studies had populations in which hepatitis virus was prevalent.

The proliferation of cholangiocytes in the intrahepatic bile duct is upregulated by oestrogens. Cholangiocytes express both oestrogens receptor-α and -β and can develop intrahepatic cholangiocarcinoma (ICC), which is the most prevalent liver malignancy after HCC [153, 154]. Laboratory findings propose that oestrogens are cofactors for cholangiocarcinogenesis [155]; in the meantime, receptor modulators can suppress advancement [156–158]. In support of hormonal contribution to carcinogenesis, studies in the general population have revealed that higher oestrogen in circulation is related to elevated odds of ICC in both men and women [159, 160]. In a meta-analysis, which comprised 12 cohorts and over a million women, prolonged (over 9 years) COC use was related to a 62% elevation in ICC risk. Interestingly, in the study, hysterectomy was associated with nearly twofold risk of ICC but not oophorectomy; this may be due to prevalently seen cofactors in this group, such as adiposity, diabetes, HRT use or misclassification of the surgical procedures in cohorts [161].

## Conclusive answers for myths

### If COCs are carcinogenic, should we use other methods primarily?

Combined oral contraceptives are safe and effective and the effects are reversible. Patients who pursue family planning should be warned of possible carcinogenic outcomes, but it should also be explained that—in addition to sexual health advantages—preferring COCs may also decrease risks for endometrial, colorectal and ovarian cancers. To this day, in no study has cancer been shown as an enhanced mortality factor among COC users.

### Can they be used by BRCA carriers and women with a family history of breast cancer?

The WHO MEC published in 2015 and US MEC for contraceptive use published in 2016 agree that the evidence to date does not propose an elevated risk for BC among women with either a family history of BC or BC susceptibility genes. Therefore, women with breast cancer susceptibility genes (such as BRCA) or a family history of BC may use COCs with safety [31, 114].

The European Society of Contraception and Reproductive Health Care published in 2018 has advocated against long-term use in BRCA carriers [162]. The UK MEC for contraceptive use (UK MEC) also take a comparable point of view placing COCs in category 3; theoretical or proven risks usually outweigh the advantages of using the method. However, it is clarified in the guideline that the very limited data in this area suggests the BC risk is not modified by COC use in both these high-risk groups [163].

BC risk elevation with COC use in this high-risk group is reported in some studies. On the other hand, others show no increase in risk. That being said, BRCA1/2 carriers should be notified that COC use may enhance BC risk. Early detection strategies for BC should be discussed with patients and periodic inspection should not be omitted; unfortunately, these strategies are not as effective for ovarian cancer and effective treatment is less likely to be achieved. The respective 5-year survival rates are 90% and 48% published by the American Cancer Society [164].

COCs can be used for contraceptive purposes in this population, but different forms of contraceptives should be elucidated. COC use for avoidance of ovarian cancer in cases where there is no need for birth control is still not advocated.

### Are modern lower-dose formulations safer than ‘old’ formulations containing higher hormone dosages?

Since the development and approval of the first COC pill, formulations have evolved from comprising high-dose oestrogen (150 mcg) to very low doses (10 and 20 mcg). Oestrogens and progestogens are among the factors that may cause BC, and this has been confirmed, for instance, in HRT in post-menopausal population. Similar concerns were raised for reproductive women using COCs, but these claims were not given much attention because the evidence was on women using high-dose formulations of old. However, some recent high-quality studies on women using modern formulations found increased BC risk [21, 165]. This new information shows that risks with COCs that contain 20–50 μg ethinylestradiol may be comparable to those found in earlier studies. This cumulative dose of oestrogen may be the major risk factor for breast cancer [166].

### Should we screen for HPV and cervical malignancy before prescribing COCs?

Screening for cervical malignancy should not be a precondition for the planning of pregnancy prevention strategies. The WHO MEC guideline acknowledges COCs as category 2 in women with HPV, CINs and cervical cancer, claiming that the advantages of COC use outweigh the risks in these groups. COCs enhance the risk with longer than 5 years of use and this enhanced risk diminishes after discontinuation and restores to those of never-users within 10 years.

Usually, cervical cancer progression is very slow, taking nearly a decade. Today, with comprehensive vaccination, widespread access to cervical cancer scanning and wider coverage of health services, elimination of cervical cancer is sought after. All women should be informed about sexually transmitted diseases, cervical cancer risk and screening strategies in polyclinic visits including family planning.

## Conclusion

For a quarter century, combined oral contraceptive carcinogenicity has been held under a microscope. Recent studies reviewed support that, in addition to their effective contraceptive effect, COCs have a strong and long-lasting suppressive effect on endometrial, ovarian and colorectal cancers. Conversely, we see that some studies report the risk of cervical and breast cancer in recent use. Although the carcinogenic effects reported on breast and cervical cancer are reversible, the overall risk can further be reduced by methods such as lifestyle changes (e.g., lactation, smoking, exercise and weight control) or HPV vaccines. In addition, since cancers are more common in advancing ages, the cumulative protective effect of drugs used in the reproductive age may become more important than the reversible effect seen in the interim period.

In the same quarter century, medical practice shunned the paternalistic approach and shifted towards co-decision-making in counselling. It is important to discuss the benefit/harm balance with patients and make a decision specific to that patient. Once the patient population that should avoid combined oral contraceptives has been identified, it is not appropriate to make decisions on the remaining women solely for fear of cancer. Up-to-date and clear information flow will both strengthen the patient–doctor relationship and increase the patient’s compliance and confidence in the method chosen.

## Conflicts of interest

The authors declare no conflicts of interest.

## Funding

This research received no specific grant from any funding agency in the public, commercial or not-for-profit sectors.

## Figures and Tables

**Table 1.** Studies reporting no increase in the risk of breast cancer associated with COC use.

	Study design	Risk analysis results
Jordan *et al* [12]	Case–Control Study	0.7% of all cases is attributable to COC use
CDC [13]	Case–Control Study	RR: 0.6–1.6 (not significant)
Nurses’ Health Study [14]	Prospective Cohort Study	>10 year COC use RR: 1.11 (95% CI: 0.94–1.32)
Marchbanks *et al* [15]	Case–Control Study	Current use; RR: 1.0 (95% CI: 0.8–1.3) Past use; RR: 0.9 (95% CI: 0.8–1.0)
Michels *et al* [16]	Prospective Cohort Study	>10 year COC use RR:1.04 (0.97, 1.11)
Oxford-FPA [5]	Prospective Cohort Study	Ever versus Never RR: 1.0 (95% CI: 0.9–1.1)
RCGP [17]	Prospective Cohort study	Ever versus Never RR: 0.98 (95% CI: 0.87–1.1)

RR: Risk Ratio; COC: Combined Oral Contraceptive; CI: Confidence Interval; CDC: Centres for Disease Control; Oxford FPA: Oxford Family Planning Association; RCGP: Royal College of General Practitioners

**Table 2.** Studies reporting an increase in the risk of breast cancer associated with COC use.

	Study design	Never vs Ever RR	Current and recent RR	Past RR (≥5 year)
RCGP [6]	Prospective Cohort Study	1.04 (99% CI: 0.91–1.17)	1.48 (99% CI: 1.10–1.97)	0.75–1.12 (No increased risk)
Mørch *et al* [21]	Prospective Cohort Study	N/A	1.19 (95% CI: 1.13–1.26)	1.05 (95% CI: 0.98–1.13)
Collaborative Group [19]	Meta-Analysis	-Prospective studies (RR ±SD : 1.07 ±0.035) -Case-control studies with population controls (RR ±SD : 1.10 ±0.081) -Case-control studies with hospital controls (RR ±SD : 1.17 ±0.035) -All studies combined (RR ±SD : 1.07 ±0.017)	-Prospective studies (RR ±SD : 1.14 ±0.091) -Case-control studies with population controls (RR ±SD : 1.16 ±0.048) -Case-control studies with hospital controls (RR ±SD : 1.18 ±0.057) -All studies combined (RR ±SD : 1.16 ±0.034)	-Prospective studies (RR ±SD : 1.14 ±0.091), -Case-control studies with population controls (RR ±SD : 1.16 ±0.048), -Case-control studies with hospital controls (RR ±SD : 1.18 ±0.057) -All studies combined (RR ±SD : 1.16 ±0.034)
Hunter *et al* [20]	Prospective Cohort Study	N/A	1.33 (95% CI: 1.03–1.73)	1.12 (95% CI: 0.95–1.33)

RR: Risk ratio; N/A: Not available; CI: Confidence interval

**Table 3.** Studies analysing the risk of colorectal cancer associated with COC use.

	Ever versus Never RR
Rennert *et al* [128]	Jews:0.49 (95% CI: 0.39–0.62) Arabs:0.14 (95% CI: 0.04–0.47)
Tsilidis *et al* [133]	0.92 (95% CI: 0.83–1.02)
Lin *et al* [132]	0.67 (95% CI: 0.50–0.89)
Charlton *et al* [130]	1.01 (95% CI: 0.91–1.12)
Iversen *et al* [6]	0.81 (95% CI: 0.66–0.99)
Kabat *et al* [131]	0.83 (95% CI : 0.73–0.94)
Gierisch *et al* [104]	0.86 (95% CI: 1.00–1.17)
Zervoudakis *et al* [134]	1.04 (95% CI: 0.93–1.16)
Brändstedt *et al* [129]	1.05 (95% CI : 0.80–1.37)
